# Interleukin-10 and tumour necrosis factor alpha promoter region polymorphisms and susceptibility to urogenital schistosomiasis in young Zimbabwean children living in *Schistosoma haematobium* endemic regions

**DOI:** 10.4102/sajid.v35i1.11

**Published:** 2020-09-03

**Authors:** Amos Marume, Arthur Vengesai, Jaclyn Mann, Takafira Mduluza

**Affiliations:** 1 Department of Infection Prevention and Control, School of Laboratory Medicine and Medical Sciences, College of Health Sciences, University of KwaZulu-Natal, Durban, South Africa; 2 Paraclinical Department, Faculty of Veterinary Sciences, University of Zimbabwe, Harare, Zimbabwe; 3 Department of Biochemistry, University of Zimbabwe, Harare, Zimbabwe

**Keywords:** cytokine polymorphisms, ssociations, *S. haematobium*, IL-10, TNF-α

## Abstract

**Background:**

Host genetic factors can influence susceptibility, morbidity and mortality from schistosomiasis. The study explored the association between single nucleotide polymorphisms (SNPs) in interleukin-10 (IL-10) and tumour necrosis factor alpha (TNF-α) promoter regions and susceptibility to *Schistosoma haematobium* infection.

**Methods:**

Urine specimens were collected from 361 primary school children aged 5–15 years from schistosomiasis endemic areas of Manicaland and Mashonaland central provinces. *Schistosoma haematobium* was diagnosed using the urine filtration method. Only 272 participants provided adequate blood for genotyping. Genotyping was performed using the amplification refractory mutation system-polymerase chain reaction. The association between IL-10 and TNF-α SNPs and *S. haematobium* infection was analysed using the chi-square test.

**Results:**

*Schistosoma haematobium* infection was confirmed in 26.8% of the participants. No significant difference in *S. haematobium* prevalence between men (51.6% of those infected) and women (48.4%) (χ^2^ = 0.008, *df* = 1, *p* = 0.928) was observed. The total IL-10 -1082 G, IL-10 -819 C and TNF-α -308G allele distribution between *S. haematobium* infected and uninfected participants was 50.7% and 51.5% (χ^2^ = 0.025, *df* = 1, *p* = 0.87), 54.3% and 60.6% (χ^2^ = 1.187, *df* = 1, *p* = 0.187) and 82.1% and 80.9% (χ^2^ = 0.099, *df* = 1, *p* = 0.753), respectively, and the differences were not significant.

**Conclusion:**

Interleukin-10 -1082 G/A, IL-10 -819 C/T and TNF-α -308 G/A SNPs were not significantly associated with susceptibility to *S. haematobium* infection. The prevalence of schistosomiasis is still in the moderate range and is similar in boys and girls.

## Introduction

Children in sub-Saharan Africa have endured many public health threats that significantly alter their prospects, health and socio-economic development. One of these main health threats, as listed by the World Health Organization (WHO), is schistosomiasis, which is caused by *Schistosoma haematobium* (urogenital) or *Schistosoma mansoni* (intestinal). These helminthic infections affect more than 200 million people worldwide and 800 million people are at risk in 76 endemic countries, leading to annual losses ranging from 1.7 to 4.5 million disability-adjusted life years.^1,2^ Some countries have made significant strides in controlling schistosomiasis (Morocco and some Caribbean countries including Sint Maarten, Saint Kitts and Vieques) and a few others have eliminated it (Japan and Tunisia).^3^ Schistosomiasis is ranked ninth as one of the most reported outpatient illnesses in Zimbabwe. The overall prevalence of *S. haematobium* and *S. mansoni* in Zimbabwe is 20.8% and 9%, respectively.^4^ Risk factors include the number of water bodies, location (rural and agricultural lands have high transmission), poverty, ignorance, age, gender, poor housing, and poor hygiene and sanitation.^5^ The disease has been linked to growth retardation, fatigue, weakness, impairment of memory and cognitive reasoning and increased risk of anaemia, leading to poor academic performance and thus limiting the potential of infected children.^3^ Irreversible damages and deaths because of kidney and/or liver damage have been reported especially in elderly populations.^1,2^ The disease has been linked to increased chances of acquiring human immunodeficiency virus (HIV); the negative consequences of schistosomiasis are even more pronounced when occurring together with other infections or diseases, for example, HIV infection, cancer and malaria.^3,6^ Chemotherapy with praziquantel (PZQ) is highly effective but re-infections are common.^1,2^ Furthermore, most people infected with schistosomiasis are asymptomatic, contributing to difficulty in controlling the disease.^3,5^

Host genetic factors, such as genetic polymorphisms altering expression of cytokines key in the Th1/Th2 differential responses, have been recognised as key influencers in parasitic infections, prognosis, morbidity, treatment outcomes and vaccine development.^7,8^ Severity of symptoms has been associated with cytokines that influence the granulomatous response, namely, interleukin-10 (IL-10) and tumour necrosis factor alpha (TNF-α). Specifically, lower IL-10 and elevated TNF-α levels are associated with an exaggerated granulomatous response to ova trapped in the bladder wall as well as other urinary tract pathologies.^9^ Other cytokines such as IL-4 and TGF-β have been associated with fibrosis after the granulomatous reactions, whilst IFN-γ has been recognised as having anti-fibrogenic effects.^10^ Interleukin-10 released by Th2 cells is recognised as an important anti-inflammatory and anti-fibrotic cytokine.^11^ Interleukin-10 elevation in the early phase of *S. haematobium* infection is linked to down-modulation of immunopathological responses and hence reduced morbidity;^12,13^ however, elevated parasite-specific IL-10 is a risk factor of re-infection.^14,15^ Low IL-6, IL-10 and TNF-α and high IL-13 levels have been linked to enhanced *S. mansoni* disease progression.^16^ However, other studies have shown that some cytokine polymorphisms, even those known to cause elevated production of TNF-α (namely at −376 and −308), have no link with major developments of hepatic periportal fibrosis (PPF) in *S. mansoni* and *Schistosoma japonicum* infections.^17^ Cytokines also influence serum Immunoglobulin E (IgE) levels, which are associated with resistance and/or susceptibility to schistosomiasis in humans.^18^ For example, immunity or resistance to schistosome infection has been associated with high and low levels of IgE and Immunoglobulin G4 (IgG4), respectively, and IL-10 alters the production of these antibodies. Interleukin-10 indirectly downregulates IL-4-induced production of IgE and directly upregulates IL-4-induced production of IgG4.^19^ Collectively, these studies have shown that IL10 and TNFα amongst other cytokines play a major role in the pathogenesis and severity of schistosomiasis, and that host immunogenetics is paramount in determining the susceptibility and/or resistance to schistosomiasis.

Given the importance of IL-10 and TNF-α in schistosomiasis, this cross-sectional study was designed to determine possible links between single nucleotide polymorphisms (SNPs) in promoter regions of *IL-10* and *TNF-α* genes and susceptibility to schistosomiasis. Given the resources, two interleukins were chosen as they could adequately represent both extremes: the pro-inflammatory (TNF-α) and the anti-inflammatory (IL-10). Interleukin-10 -1082 G/A, IL-10 -819 C/T and TNF-α -308 G/A were chosen because they were associated with other infectious and non-infectious diseases.^18,20^ To this end, we performed genotyping of cytokines IL-10 and TNF-α as well as confirmed the presence or absence of *S. haematobium* infection, the most prevalent schistosome infection, in 361 children in endemic areas of Zimbabwe.

## Materials and methods

### Study population and sampling

The study population was previously described by Midzi et al.^21^ Primary school children aged between 6 and 15 years were targeted and recruited for the study as they constitute the high-risk age group for schistosomiasis.^21^ Briefly, Manicaland and Mashonaland central provinces in rural Zimbabwe were selected for this study based on their geographical locations (characterised by high annual rainfall and open water bodies, conditions conducive for *Schistosoma* species leading to high schistosomiasis endemicity) and relatively higher prevalences in previous studies.^21^ Simple random sampling method was used to select schools per province, Bandanyenje primary school in Manicaland and Bemberi primary school in Mashonaland, using the lottery method. The sample size of 361 for parasitology and 272 for genotyping (some were not willing or able to provide adequate blood sample for genotyping) was calculated using the EPI Info 6 statistical package as previously described by Midzi et al.^21^ Briefly, based on the national primary school enrolment of more than 2 million children, the national sample size calculated by Epi Info version 6 was 15 818 as of 2014.^21^ The calculated sample size per school was 50 children. The researchers targeted 100 children to cater for errors and the fact that some children were unwilling and/or unable to provide enough blood sample for genotyping. The sample size was higher than what was anticipated because of overwhelming responses.

### Cytokine genotyping

Deoxyribonucleic acid (DNA) for the genotyping was extracted from approximately 300 µL of whole blood using the Qiagen FlexiGene DNA extraction kit, following the manufacturer’s protocol. Interleukin-10 and TNF-α promoter region SNPs were genotyped using amplification refractory mutation system-polymerase chain reaction (ARMS-PCR). Two different primers (Inqaba Biotechnology, Pretoria, South Africa; Table 1), specific for wild-type genotype (1 µL) and mutant genotype (1 µL), respectively, were separately mixed with 1 µL (10 mM) generic primer, 0.5 µL 10 mM forward and 0.5 µL 10 mM reverse internal control primers (human growth hormone), 12.5 µL quick load Taq 2x master mix (New England Biolabs, Ipswich, MA) and 4.5 µL of sterile, nuclease-free water (New England Biolabs, Ipswich, MA). About 5 µL of the template DNA was added to the mastermix prior to loading onto a thermocycler (PXE 0.2 thermocycler, Thermo Electron Corporation, Waltham, MA). Interleukin-10 -1082 G/A and IL-10 -819C/T alleles were amplified using the following conditions: 1 min denaturation step at 95 °C; 10 cycles of 15 s at 95 °C, 50 s at 65 °C and 40 s at 72 °C; 20 cycles of 20 s at 95 °C, 50 s at 59 °C and 30 s at 72 °C, followed by cooling at 4 °C.

**TABLE 1:** Wild-type, mutant primer and generic primer sequences for the determination of human interleukin-10 -1082, interleukin-10 -819 C/T and tumour necrosis factor-alpha -308 G/A promoter region polymorphisms.

Cytokine	Position	Mutation	Primer
IL-10	−1082	A→G	Wild type: TAAGGCTTCTTTGGGAG Mutant: TAAGGCTTCTTTGGGAA Generic: TAAATATCCTCAAAGTTCC
IL-10	−819	C→T	Wild type: CCCTTGTACAGGTGATGTAAC Mutant: CCCTTGTACAGGTGATGTAAT Generic: AGGATGTGTTCCAGGCTCCT
TNF-α	−308	G→A	Wild type: AGGTTTTGAGGGGCATTG Mutant: AGGTTTTGAGGGGCATGA Generic: CAGCGCAAAACTTCCTTGGT

IL-10, interleukin-10; TNF-α, tumour necrosis factor alpha.

**FIGURE 1:**
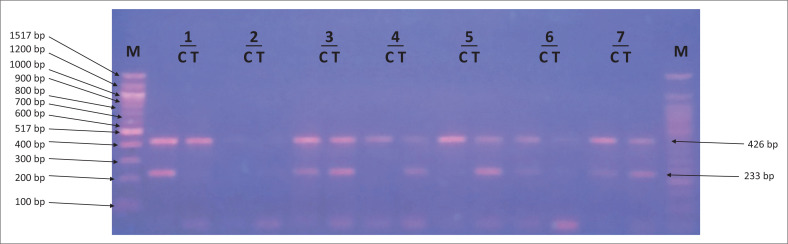
Amplicons for interleukin-10 -819 C/T single nucleotide polymorphism. Lane M shows 100 base pair (bp) molecular marker. The 426 bp fragments correspond to the internal control (the human growth hormone gene). The 233 bp fragments were specific for C and T alleles of IL-10 -819 C/T single nucleotide polymorphisms. Lanes labelled 3, 6 and 7 show heterozygous CT genotypes. Lanes labelled 1 and 4 show homozygous CC and TT genotypes, respectively.

**FIGURE 2:**
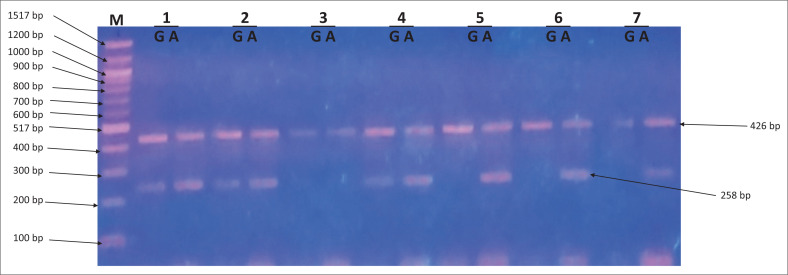
Amplicons for interleukin-10 -1082 G/A single nucleotide polymorphism. Lane M shows 100 base pair (bp) molecular marker. The 426 bp fragments correspond to the internal control. The 258 bp fragments were specific for G and A alleles of interleukin-10 -1082 G/A single nucleotide polymorphisms. Lanes labelled 1, 2 and 4 show heterozygous GA genotype. Lanes labelled 5, 6 and 7 show homozygous AA genotype.

**FIGURE 3:**
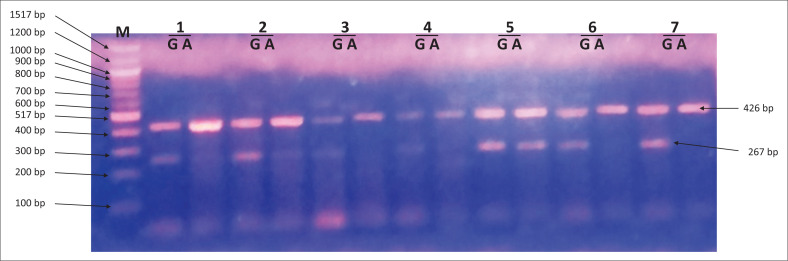
Amplicons for tumour necrosis factor-alpha -308 G/A single nucleotide polymorphism. Lane M shows 100 base pair (bp) molecular marker. The 426 bp fragments correspond to the internal control. The 267 bp fragments were specific for G and A alleles of tumour necrosis factor-alpha -308 G/A single nucleotide polymorphisms. Lane 5 shows heterozygous GA genotype. Lanes labelled 6 and 7 show homozygous GG genotype.

Amplification refractory mutation system-polymerase chain reaction amplicons were analysed on a 2% agarose gel to score the presence or absence of the cytokine gene polymorphisms (Figures 1–3).

### Detection of *Schistosoma haematobium*

*Schistosoma haematobium* was diagnosed by the microscopic examination of urine specimens for the presence of parasite eggs using the urine filtration technique. In brief, urine specimens were collected from willing participants and 10 mL of urine was filtered through a nitrile filter membrane. The membrane was stained with iodine and examined using a light microscope. Participants who were *S. haematobium* positive were treated with a single dose of PZQ (40 mg/kg of body weight). Bread and orange juice were given as supplementary food to enhance the absorption and nauseating effects of PZQ.

### Statistical analysis

The allele frequencies and genotype distribution of *S. haematobium-*infected and uninfected participants were then analysed using the chi-square test. All analyses were performed using Statistical Package for the Social Sciences (SPSS) version 21 and *p*-values <0.05 were considered statistically significant.

### Ethical consideration

Blood and urine specimens were obtained from the study participants following the signing of informed consent forms by their parents or guardians to allow their participation. The study was registered and ethically approved by the Zimbabwe’s ethics board for biomedical research (the Medical Research Council of Zimbabwe – MRCZ/A/1710). In addition, community leaders, the Provincial Medical Director, the District Medical Officer and education directors also granted permission to conduct the study at Bemberi and Bandanyenje primary schools. The children and parents were informed about the aims, risks and benefits of the study. Participation was voluntary and participants were free to withdraw from the study at any time.

## Results

The demographics of the study participants are summarised in Table 2.

**TABLE 2:** Summary of study population.

Study area	Age range	Sex				Total population	
		Male		Female			
		%	*n*	%	*n*	%	*n*
Bemberi	6–15 years	48.2	63	50.8	65	35.4	128
Bandanyenje	7–14 years	51.6	118	49.4	115	64.6	233

*n*, number of participants.

### *Schistosoma haematobium* prevalence

Amongst the 361 study participants from Bemberi and Bandanyenje, overall 26.8% (28.9% and 25.8%, respectively) were found to be infected with *S. haematobium*. No significant difference was observed between the prevalence of men infected with *S. haematobium* (51.6%) and that of women infected with *S. haematobium* (48.4%, χ^2^ = 0.008, *df* = 1, *p* = 0.928).

#### Distribution of the interleukin-10 -1082, interleukin-10 819 and tumour necrosis factor-α -308 genotypes and alleles frequencies in uninfected and infected *Schistosoma haematobium* groups in Bemberi

Total genotype and allele frequencies of IL-10 -1082 G/A, IL-10 -819 C/T and TNF-α -308 G/A in Bemberi are shown in Table 3. The IL-10 -1082 G and A alleles were evenly represented in the population (51.7% and 48.3%, respectively) and occurred with similar frequency in *S. haematobium-*infected and uninfected participants. The frequency of IL-10 -819 C allele was lower in *S. haematobium-*infected participants (50%) compared to *S. haematobium*-uninfected participants (56.8%), and correspondingly, the IL-10 -819 T allele was higher in infected participants (50%) compared to uninfected participants (43.2%); however, these differences were not statistically significant (*p* = 0.342). No homozygosity for the alleles IL-10 -1082 A and TNF-α A was observed in this study population. Tumour necrosis factor-α -308 G/A alleles and genotypes had the same frequency in *S. haematobium-*infected and uninfected participants; however, the major G allele was almost four times more prevalent than the minor A allele. Interleukin-10 -1082 G/A, IL-10 -819 C/T and TNF-α -308 genotypes were examined using the chi-square test to establish whether each was associated with susceptibility to *S. haematobium* infection. However, the distribution of genotype frequencies did not differ significantly between *S. haematobium*-infected and uninfected participants (see Table 3).

**TABLE 3:** Distribution of the interleukin-10 -1082, interleukin-10 819 and tumour necrosis factor alpha genotypes and alleles frequencies in uninfected and infected *Schistosoma haematobium* groups in Bemberi.

Cytokine polymorphism	Allele or genotype	Frequency						*p*	χ^2^	*df*
		Uninfected		Infected		Total population				
		%	*n*	%	*n*	%	*n*			
IL-10 -1082 G/A	G	51.7	31	51.5	34	51.7	125	0.979	0.001	1
	A	48.3	85	48.5	32	48.3	117	-	-	-
	GG	3.4	3	6.1	2	4.1	5	0.514	0.426	2
	GA	96.6	85	93.9	31	95.9	116	-	-	-
	AA	0	0	0	0	0	0	-	-	-
IL-10 -819C/T	C	56.8	100	50	33	55	133	0.342	0.901	1
	T	43.2	76	50	33	45	109	-	-	-
	CC	23.9	21	12.1	4	20.7	25	0.364	2.021	2
	CT	65.9	58	75.8	25	68.6	83	-	-	-
	TT	10.2	9	12.1	4	10.76	13	-	-	-
TNF-α -308 G/A	G	89.2	157	89.4	59	89.3	216	0.966	0.002	1
	A	10.8	19	10.6	7	10.7	26	-	-	-
	GG	78.4	69	78.8	26	78.5	95	0.964	0.002	1
	GA	21.6	19	21.1	7	21.5	26	-	-	-
	AA	0	0	0	0	0	0	-	-	-

IL-10, interleukin-10; TNF-α, tumour necrosis factor alpha; df, degrees of freedom.

#### Distribution of interleukin-10 -1082, interleukin-10 -819 and tumour necrosis factor-α -308 genotypes and alleles frequencies in *Schistosoma haematobium* infected and uninfected groups in Bandanyenje

Total genotype and allele frequencies in Bandanyenje are shown in Table 4. The IL-10 -1082 G/A allele frequencies were evenly distributed, where 51% had the G allele and 49% had the A allele. There was no significant difference (*p* = 0.154) in the genotype frequency distribution for IL-10 -1082 GA between *S. haematobium-*infected (100%) and *S. haematobium*-uninfected (94.7%) participants. Only 5.3% *S. haematobium*-uninfected participants had the GG genotype and the AA genotype was absent in both groups. Statistical analyses demonstrated that the difference in the frequency of the IL-10 -819 T/C alleles was not significant between *S. haematobium*-infected and uninfected participants (*p* = 0.397). Likewise, there was no statistical difference in the distribution of IL-10 -819 T/C genotype frequencies between *S. haematobium*-infected and uninfected participants (*p* = 0.183). The percentage frequencies of TNF-α -308 G allele (74.6% compared to 75.7%) and the TNF-α GG genotype (53.5% compared to 54.1%) were similar in *S. haematobium*-uninfected and infected participants, respectively (*p* = 0.901). Likewise, there were no significant differences in the distribution of the TNF-α A allele in *S. haematobium-*infected and uninfected participants (*p* = 0.848).

**TABLE 4:** Distribution of the interleukin-10 -1082, interleukin-10 819 and tumour necrosis factor alpha genotypes and alleles frequencies in uninfected and infected *Schistosoma haematobium* groups in Bandanyenje.

Cytokine polymorphism	Allele or genotype	Frequency						*p*	χ^2^	*df*
		Uninfected		infected		Total population				
		%	*n*	%	*n*	%	*n*			
IL-10 -1082 G/A	G	51.3	117	50	37	51	154	0.844	0.039	1
	A	48.7	111	50	37	49	148	-	-	-
	GG	5.3	6	0	0	4	6	0.154	2.028	2
	GA	94.7	108	100	37	96	145	-	-	-
	AA	0	0	0	0	0	0	-	-	-
IL-10 -819C/T	C	63.6	145	58.1	43	62.3	188	0.397	0.716	1
	T	36.4	83	41.9	31	37.7	114	-	-	-
	CC	31.6	36	21.6	8	29.1	44	0.183	3.392	2
	CT	64	73	73	27	66.2	100	-	-	-
	TT	4.4	5	5.4	2	4.5	7	-	-	-
TNF-α -308 G/A	G	74.6	170	75.7	56	74.8	226	0.848	0.037	1
	A	25.4	58	24.3	18	25.2	76	-	-	-
	GG	53.5	61	54.1	20	53.6	81	0.901	0.209	2
	GA	42.1	48	43.2	16	42.4	64	-	-	-
	AA	4.4	5	2.7	1	4	6	-	-	-

IL-10, interleukin-10; TNF-α, tumour necrosis factor alpha; df, degrees of freedom.

#### Distribution of the interleukin-10 -1082, interleukin-10 819 and tumour necrosis factor-α genotypes and alleles frequencies in uninfected and infected *S. haematobium* groups in the total study population

The total genotype allele frequencies and genotype frequencies of IL-10 -1082 G/A, IL-10 -819 C/T and TNF-α -308 G/A in total study population are shown in Table 5. Overall, there were no statistically significant differences in IL-10 and TNF-α wild-type allele distribution between *S. haematobium*-infected and uninfected participants. Interleukin-10 -1082 G allele, IL-10 -819 C allele and TNF-α -308 G allele and distributions between *S. haematobium*-infected and uninfected participants were 50.7% and 51.5% (*χ*^2^ = 0.025, *df* = 1, *p* = 0.87), 54.3% and 60.6% (χ^2^ = 1.187, *df* = 1, *p* = 0.187) and 82.1% and 80.9% (χ^2^ = 0.099, *df* = 1, *p* = 0.753), respectively. Similarly, there were no significant differences in IL-10 and TNF-α mutant allele distribution between *S. haematobium*-infected and uninfected participants. There was no significant difference in the genotype frequency distribution for IL-10 -1082 GA between *S. haematobium-*infected (97%) and *S. haematobium*-uninfected (95.5%) participants (*p* = 0.154). Only 4% of participants had the GG genotype which is associated with high levels of IL-10 and the AA genotype associated with low levels of IL-10 was absent in both groups. Statistical analyses also demonstrated that the difference in the frequency of the IL-10 -819 T/C genotypes was not significant between *S. haematobium*-infected and uninfected participants (*p* = 0.183). The TNF-α -308 GG genotype had the highest distribution (64.7%) followed by the GA genotype (33.1%), and the homozygous AA genotype had the lowest distribution (2.2%). However, there were no statistical differences in the distribution of TNF-α -308 G/A genotypes between *S. haematobium*-infected and uninfected participants (*p* = 0.872).

**TABLE 5:** Distribution of the interleukin-10 -1082, interleukin-10 819 and tumour necrosis factor alpha genotypes and alleles frequencies in uninfected and infected *Schistosoma haematobium* groups in the total study population.

Cytokine polymorphism	Allele or genotype	Frequency						*p*	χ^2^	*df*
		Uninfected		Infected		Total population				
		%	*n*	%	*n*	%	*n*			
IL-10 -1082 G/A	G	51.5	208	50.7	71	51.3	279	0.87	0.025	1
	A	48.5	196	49.3	69	48.7	265	-	-	-
	GG	4.5	9	2.9	2	4	11	0.559	0.342	1
	GA	95.5	193	97	68	96.6	261	-	-	-
	AA	0	0	0	0	0	0	-	-	-
IL-10 819 C/T	C	60.6	100	54.3	33	59	133	0.187	1.187	1
	T	39.4	159	45.7	64	41	223	-	-	-
	CC	28.2	57	17.1	12	25.4	69	0.183	3.392	2
	CT	64.1	131	74.3	52	67.3	183	-	-	-
	TT	6.9	14	8.6	6	7.4	20	-	-	-
TNF-α 308 G/A	G	80.9	327	82.1	115	81.2	442	0.753	0.099	1
	A	19.1	77	17.9	25	18.8	102	-	-	-
	GG	64.4	130	65.7	46	64.7	176	0.872	0.275	2
	GA	33.2	67	32.9	23	33.1	90	-	-	-
	AA	2.5	5	1.4	1	2.2	6	-	-	-

IL-10, interleukin-10; TNF-α, tumour necrosis factor alpha; df, degrees of freedom.

## Discussion

Urogenital schistosomiasis (caused by *S. haematobium*) remains a significant threat especially for children in Zimbabwe. Host genetic factors can influence susceptibility to and severity of schistosomiasis; however, there is significant lack of studies investigating this in Zimbabwe. This study investigated the prevalence of schistosomiasis in known endemic areas of Manicaland and Mashonaland central provinces in rural Zimbabwe. It also investigated possible links between polymorphisms in the promoter regions of cytokines IL-10 and TNF-α and susceptibility to schistosomiasis, because previous studies in other parts of the world have implicated those polymorphisms in the disease susceptibility and/or immunity.

This study highlighted the importance of the disease as it established a prevalence of 26.8%, which is well above the national prevalence of 20.8% in Zimbabwe in 2012.^4^ The findings may suggest a low success of current control and treatment programmes, poor coverage or the study areas being part of the remaining urogenital schistosomiasis hotspots in Zimbabwe. Similar prevalences in both boys and girls suggest overlapping unsafe water-related chores and/or activities.

Susceptibility to schistosomiasis has been associated with genetic components, apart from socio-economic, environmental and ecological factors.^22^ Thus, certain genotypes are thought to be more prone to schistosomiasis than others. The genes *TNF-α* and *IL-10* have attracted considerable attention as possible contributors to susceptibility or resistance to infectious, non-communicable, immune-mediated and autoimmune diseases.^20^ Studies have demonstrated an association between different allelic variants and differential production of IL-10. Polymorphism of the promoter region has led to different haplotypes with different levels of IL-10 production, that is, ‘high’ IL-10 producer haplotype (GCC/GCC), ‘intermediate’ producer haplotypes (GCC/ACC, GCC/ATA) and ‘low’ producer haplotypes (ATA/ATA, ACC/ATA, ACC/ACC), where positions 1082, 819 and 592 are represented.^18^ Overall, high production is hinged on having a G in the -1082 position independent of the -819 and -592 polymorphisms.

High IgE and low IgG4 antibodies against a variety of schistosome antigens have been associated with resistance. Immunoglobulin G4 (IgG4) is thought to inhibit the actions of IgE. Interleukin10 is associated with IgG4 production and blocking the receptors of IL10 in experimental mice models has been linked to the development of significant protection against re-infection after treatment.^18,23^ Interleukin10, predominantly produced by CD4+ lymphocytes, obstructs the development of acquired resistance, reduces morbidity and prolongs survival in schistosomiasis.^12,15^ The ‘low’ ATA/ATA haplotype has been associated with increased circulating eosinophil counts; worm-specific IgG1, IgG2b and IgE levels; and enhanced Th1, Th2 and Th17 responses, which are key in conferring immunity against schistosomes.^15,18^ Lower levels of IL10 thus correspond to low susceptibility to schistosomiasis and development of immunity after treatment. After correction for sex, age and infection status at study onset, high levels of parasite-specific IL-10 were recognised as a risk factor for re-infection in a study conducted in Gabon.^14^

Tumour necrosis factor alpha, a potent immunomediator and pro-inflammatory cytokine, is generally produced in the early inflammatory stages of infection. It has been linked with liver granulomas/fibrosis and egg-laying of the parasite.^24,25^ Tumour necrosis factor alpha and INF-γ are indicators of a Th1 response and are often elevated in acute schistosomiasis. Contradictions can be observed from scientific reports as some conclude that TNF-α is protective against severe disease, whilst others noted that high levels aggravate disease in *S. mansoni*.^16^ The increased frequency of the rare allele TNF-308A (TNF2) has been reported in autoimmune disorders, such as rheumatoid arthritis, systemic lupus erythematous and coeliac disease. Relative to the more common TNF1 (TNF-308G) allele, the TNF2 allele is a more powerful transcriptional activator, and thus more TNF-α is expected in individuals with TNF2 allele(s).^20^ The TNF2 allele can then be hypothesised as increasing the resistance of the host to local infection (by increasing local production of TNF at the infection site). The limiting of successful infection after entry of cercariae, is also associated with increased risk for severe pathology and other chronic, inflammatory or autoimmune diseases.^26^ In Kenya, TNF-308 promoter polymorphism allele 2 has been associated with early childhood mortality and malaria morbidity. Tumour necrosis factor 2 has also been associated with pathogenesis of asthma, peptic or duodenal ulcers, coronary heart disease and angina.^27^

We therefore hypothesised that the IL-10 -1082 A allele and TNF-α 308 A allele, as well as genotypes with these alleles, would be associated with reduced susceptibility to schistosomiasis in our study population. The frequencies of IL-10 -1082 and TNF-α 308 alleles or genotypes were similar to those observed in other studies. The frequency of G allele (TNF-α 308 G/A) was above 80% as was expected in African people.^26^ In addition, IL-10 -1082 G/A genotypes with at least one G had similar frequencies as was observed in other studies.^28,29^ However, contradictory to our initial hypothesis of these genotypes associating with susceptibility to schistosomiasis, we observed no difference in frequency of these genotypes between *S. haematobium*-infected and uninfected participants. The findings further highlight the complexities in host immune–parasite interactions. As the study was not controlled, many confounding and predisposing factors (such as poverty, possible under-nutrition, high prevalence, high-contaminated water contact, co-infections amongst other risk factors) might have eroded the effects of host genetic factors that were expected. Studies have shown that schoolgoing children are at a higher risk than other age groups.^30^ In the same study, Ismail et al.^30^ also demonstrated that high water contact associated with communal and agricultural (irrigation) lands was linked with high prevalence rates.^30,31^ Other factors associated with prevalence and high infection intensities in endemic areas are gender, occupation, female household head’s education level, religion, socio-economic status and house location.^32^ The highlighted factors have been found to influence a person’s contact with infested water.^32^ Thus, the high prevalence noted and the lack of proper control for the highlighted factors in this study could have eroded the minor effects of host genetic factors. There is probably a need to have larger sample sizes and correction for the suggested confounding and predisposing factors in future studies. Controlled studies may also highlight the link between IL-10 production levels to susceptibility to schistosomiasis. The study was also limited by the number of children who were willing or able to provide adequate blood sample for genotyping.

## Conclusion

The findings failed to demonstrate any significant relationship between host genetic factors considered (i.e. IL-10 -1082 G/A, IL-10 -819 C/T and TNF-α -308 G/A SNPs) with susceptibility to urogenital schistosomiasis. The prevalence of schistosomiasis is still in the moderate range and is similar in boys and girls. Future studies are recommended to include investigations of possible links between the SNPs and morbidity or pathology.
